# European Practice Assessment of Cardiovascular risk management (EPA Cardio): protocol of an international observational study in primary care

**DOI:** 10.1186/1748-5908-4-3

**Published:** 2009-01-07

**Authors:** Michel Wensing, Sabine Ludt, Stephen Campbell, Jan van Lieshout, Eckhard Volbracht, Richard Grol, on behalf of the EPA Cardio Project Group

**Affiliations:** 1 Radboud University Nijmegen Medical Centre, Scientific Institute for Quality of Healthcare, Germany; 2 Heidelberg University Hospital, Department of General Practice and Health Services Research, Germany; 3 University of Manchester, National Centre for Primary Care Development and Research, UK; 4 Bertelsmann Foundation, Gütersloh, Germany

## Abstract

**Background:**

Despite important improvements in available prevention and treatment, cardiovascular diseases (CVD) remain an important cause of morbidity and mortality. Not all high-risk patients and patients with CVD have healthy lifestyles and receive the best possible healthcare. Internationally comparative data are needed to compare cardiovascular risk management in different countries, and to examine the impact of improvement programs and others factors.

**Objectives:**

This study aims to provide internationally comparative data on cardiovascular risk management provided in primary care and on health-related lifestyles of patients in Europe. The study will also explore the views of doctors and patients on innovative preventive services for CVDs.

**Design and methods:**

An observational cross-sectional study is planned. In 10 European countries, stratified samples of 36 practices per country will be recruited. In each practice, three samples of 15 patients each will be sampled: patients with coronary heart disease, patients at high risk for CVD, and healthy adult patients. The quality of cardiovascular risk management has been specified in terms of 44 performance indicators that resulted from an international Delphi-procedure with general practitioners. Most indicators are based on medical records, and some on a structured interview with a contact person of the practice. Lifestyle (smoking, physical exercise, diet) will be measured with previously validated questionnaires that are completed by patients. Additional measures include practice characteristics and exposure to programs to improve cardiovascular care.

## Background

Cardiovascular diseases (CVD) have a major impact on the mortality and quality of life of human populations across the world, despite improvements in lifestyle and innovations in the prevention and treatment of CVD in previous decades [1]. Cardiovascular risk management includes the clinical management of established CVD, prevention of CVD in patients at high risk for developing CVD, and improvement of health-related lifestyles in the population [2]. Though numbers of deaths from coronary heart disease (CHD) in developed countries have declined [3] and cardiovascular care may have improved in recent years [4], many eligible individuals currently still do not receive the best available treatment and prevention for CVD [5]. A better insight into cardiovascular risk management in primary care and health-related lifestyles of patients could help to develop effective programs for improving current practice.

Data on current cardiovascular risk management and patients' lifestyles are needed, both to identify performance gaps and set specific targets for improvement, and to identify underlying factors and tailor interventions to relevant barriers for change. We do not have specific hypotheses on the quality of cardiovascular risk management or patients' lifestyles, except that we expect much variation across patients and practices within each of the countries. Inadequate delivery of cardiovascular risk management may be related to various factors. For instance, it may be related to inadequate perception of cardiovascular risk by physicians [4], as well as to concerns about the efficiency and ethical implications of providing cardiovascular prevention to individuals at low risk for developing CVD [6]. The clinical benefits and efficiency of primary and secondary prevention are continued topic of scientific debate [7]. Alternatively, it may be related to organizational and financial barriers in practice organizations for providing cardiovascular risk management. For instance, organizational characteristics of general practices, such as size of scale and teamwork, proved to be associated with providing cardiovascular risk management [8].

While data on cardiovascular risk management are available in a number of countries, our planned study aims at providing internationally comparative data. Such data have a potential advantages compared to national data. Trends and associations identified in international datasets may be more robust for the confounding influence of national healthcare systems and national cultures. For instance, studies in different European studies showed across these countries that patient evaluations of accessibility were most positive in small general practices [9], and that physician workload per 1,000 patients was consistently lower in larger practices [10]. The consistency of these findings makes it more likely that the associations were not confounded by characteristics of a specific healthcare system or national culture. Furthermore, international comparison of performance between different countries can stimulate stakeholders for improvement, although country differences can often be attributed to many other factors than those of interest.

Our focus is on primary care, because a substantial part of prevention and chronic care for CVD is delivered in this sector. Many countries have large-scale programs to improve cardiovascular risk management in primary care, such as disease management programs in Germany [11], indicator-based incentive contracts in the United Kingdom [3], and practice support in outreach visits in The Netherlands [12]. Interestingly, these programs tend to focus either on risk management in patients with established CVD in some countries, and on lifestyle education for the population in other countries [13]. An important question is what impact exposure of a practice to these programs has on the quality of cardiovascular risk management. Internationally comparative data can enable a comparison of programs across countries.

## Aims and objectives

This study protocol concerns an international study of cardiovascular risk management in primary care in Europe. Its overall aim is to provide insight into the current services delivered in primary care to prevent CVD, with the aim to inform and support primary care practices as well as national health policies and decision makers in this domain. Appendix 1 provides definitions of key concepts in this study protocol.

Key objectives are:

1. To describe the quality of cardiovascular risk management services provided to patients with established CHD and to patients with high risk for developing CVD in primary care using performance indicators, and to compare countries in these domains.

2. To describe specific aspects of health-related lifestyle (smoking, physical exercise, diet) in high risk patients and in healthy patients in general practice across Europe.

3. To determine the association between exposure of a practice to quality improvement programs and the quality of cardiovascular risk management and patients' lifestyles.

4. To identify associations of the quality of cardiovascular risk management provided and characteristics of patients, health professionals, primary care practices, and countries with different health care systems.

5. To describe the experiences and views of general practitioners (GPs) and healthy adults on what innovative services a general practice could provide regarding primary prevention of CVD.

## Hypotheses

While the study is mainly descriptive and explorative (aimed at generating hypotheses), we tentatively formulated the following hypotheses:

Hypothesis one: The quality of cardiovascular risk management shows variation across countries, general practices, and patients (differences of 15% or more on performance indicators).

Hypothesis two: Better cardiovascular risk management is associated with the following practice characteristics:

1. More structured practice management, including implementation of information technology, organisation of chronic care and prevention, and structured quality improvement.

2. Involvement of more health professions in the practice in providing cardiovascular risk management and in preventive activities in general.

3. Larger practice size, because of size of scale advantages.

4. More exposure to and engagement of the practice in cardiovascular quality improvement projects and continuing education by health professionals on cardiovascular care.

Hypothesis three: At a country level, better quality of cardiovascular risk management in patients with CHD is associated with:

1. A stronger primary care system in the country

2. Nationwide programs to improve cardiovascular risk management in general practice

## Design and methods

The study has a cross-sectional observational design. It is internationally comparative (focused on description and comparison of countries) and explorative (focused on factors in patients, professionals and practices associated with outcomes). Ethical approval for the study will be sought in each of the participating countries, according to national laws and regulations.

### Study populations

The study includes patients, health professionals, and general practices in different countries.

### Countries

We include 10 countries: Austria, Belgium, England, Finland, France, Germany, Netherlands, Slovenia, Spain, and Switzerland. This is a comprehensive sample of countries in North, West, South and Central Europe. Although all these countries have primary care practices, there is substantial heterogeneity regarding the position of primary care in the healthcare system. For instance, primary care physicians coordinate access to specialized medical care only in some countries, while medical specialists can be consulted directly in other countries.

### Practices

Stratified random sampling of 36 practices per country is planned. This sample size was chosen because it is feasible in the context and budget of this project, while experience showed that it has been large enough to give robust results and can be considered reasonably representative for a country. A general practice is the smallest organisational unit, in which primary care physicians are based in their daily work to provide care to patients. The practice may be part of a larger organisational network, such as a multidisciplinary health centre or primary care trust (for instance to share patient lists, financial risk, legal accountability, support staff, etc.). This wider organisational context is not considered in the sampling in this project.

We aim to select representative samples of practices per country. Random sampling would be the best method, but the sample is not likely to be representative if only a small minority of randomly sampled practices accept the invitation to participate in the study. It was considered to be important that the sample of practices roughly represents the national situation as closely as possible, both for the generalisability of our findings and for drawing policy implications. Country partners are instructed to avoid recruiting only a special type of practice, such as training practices, academic practices, or practices in a special local network.

We aim for a stratified random sample, using two factors internationally for stratification: practice size and urbanisation (See Table 1). The assumption is that these factors are prognostic for our main measures of cardiovascular risk management – although strong research evidence to support this claim is lacking. The definition is as follows:

**Table 1:** International stratification scheme.

Practice size	Population density	
	Rural/town	Urban

Small	A	C

Large	B	D

### Practice size

Small practice size is defined as up to two full-time equivalent (FTE) primary care physician, while large practices have more than two FTE primary care physicians (regardless of their type of contract and reimbursement; but excluding trainees and nurse practitioners)

### Urbanisation

Urban is defined as more than 100,000 inhabitants; rural or town is defined as less than 100,000 inhabitants (considering the geographical location of the practice, although the patients may come from other areas).

The actual numbers in the cells (Table 1A, B, C, and 1D) are to reflect each county's national situation as much as possible, even if this means that in some countries some cells have few or no practices. For example, if a country has no large practices, cells B and D would be empty in that country. Country project partners are instructed to develop additional criteria for stratifying the sample in their country according to practice size and urbanization, particularly if some cells in the international stratificiation table were empty. For example, if most practices are larger than two FTE GPs, two strata within the larger practices may be defined (*e.g*., up to four FTE GPs versus five FTE GPs or more). Project partners are asked to provide information on the planned and actual stratification table for their country.

Within each of the strata, each country partner is asked to sample randomly from a regional or national list of practices. In other words, if a practice declines, a similarly sampled practice from the same stratum will be approached. For logistical reasons it is acceptable to sample in one or a few geographical areas in a country. The degree to which these regions represent the country as a whole will be described qualitatively in terms of health system and population health.

### Health professionals

The study considers all staff physically working in each general practice, including physicians, nurses (nurse practitioners, practice nurses, specialised nurses, psychiatric nurses etc.), practice assistants (whether or not with clinical tasks), allied health professionals (physiotherapists etc.), psychologists, midwives, physician assistants, administrative people, and managers. The staff may be employed by the practice or by another organisation (*e.g*., nurses in the practice, who are employed by mental health organisation). The study excludes staff working in the same health centre, or other larger organization, but based in a different practice.

### Patients

The study is focused on three patient samples, which will be identified in each of the participating practices:

Patients with established CHD. This includes myocardial infarction, angina pectoris, or vascular surgery (diagnoses based on medical records at the general practice). Patients with established diabetes are excluded to enhance the homogeneity of the study population.

Patients with high risk for developing CVD. This includes meeting one of the following criteria: 10% CVD mortality risk or 20% CVD morbidity risk in 10 years, ideally based on an individual risk assessment using validated CVD risk tables. If this is not available, we defined a proxy measure: presence of three out of the following four risk factors: hypertension, hypercholesterolemia, smoking, men over 60 years (cut-off points as defined nationally). Patients with established diabetes or established CVD are excluded from this group.

Patients aged 18 to 45 years (unselected), registered or regular visitors in the practice. The underlying argument for focusing on this age group is that we assume that health behaviors at younger age tend to be continued at later age.

Exclusion criteria for all patient samples are: terminal illness, cognitive impairment, psychiatric illness, and poor language skills.

### Procedures

For the first two patient samples (CHD patients and high risk patients) we plan to collect data from medical records and from patient questionnaires. Depending on the national context and regulations, different procedures may be used. In countries where informed consent is requested, 30 patients will be sent the questionnaire with an informed consent form for abstracting medical record data, expecting at least 50% informed consent forms will be returned. Then, data are abstracted from those patients' medical records. If no informed consent is required, a sample of 15 patients will be identified for data-extraction from their medical records. A larger sample of patients (n = 30), including these 15 patients, will be identified and sent questionnaires. These procedures were tested in a pilot studies in some general practices in each of the countries, and the results were discussed in a plenary international meeting of all researchers in order to standardize the sampling procedures as much as possible.

For the third sample, adults 18 to 45 years, we will take a random sample of a list of patients registered at the practice. In countries where there is no patient registration, alternative methods will be based on a sample of patients taken from a list; *e.g*., by taking every second or third patient until 40 patients are selected.

### Accuracy of figures

The accuracy of the figures is focused on the confidence interval associated with mean values rather than on differences between countries, practices, or subgroups. We will aim for high accuracy of the figures per country, and will use n = 36 practices per country as the maximum feasible number. We estimate an average score of 65% in a specific country, *e.g*., 65% of patients receive care according to a specific indicator (dichotomous outcome) or 65% of the maximum score on a continuous outcome (*e.g*., a five-point answering scale). A design effect based on ICC = 0.05 is assumed. Power = 0.80 and alpha = 0.05. We will aim for 95% CI interval of 60 to 70%, so we will need 493 patients per country (n = 14 per practice). For each indicator and measure data will be collected from 15 patients (from records or questionnaires) per practice. The actual number of patients approached depends on the procedure and the expected response rate, and may therefore vary across countries.

### Measures

The following measurement methods will be used: medical record audit, patient questionnaires, and a questionnaire and an interview guide for a contact person in each practice. Specific measures include (see also Table 2.):

**Table 2:** Measurement domains and data collection methods

	Data-abstraction from sampled medical records	Interview and questionnaire for contact persons in the practice	Survey in CHD patients	Survey in high risk patients	Survey in healthy patients
1. Clinical and organisational performance in cardiovascular prevention (= EPA Cardio instrument)	X	X			

2. Engagement of the practice in quality improvement projects		X			

3 Practice characteristics, including EPA dimensions		X			

4a Patient demographics and chronic diseases			X	X	X

4b PACIC			X		

4c. EQ-5D			X	X	

4d EUROPEP			X	X	X

4e Lifestyle: smoking, physical exercise, diet				X	X

5 Views on primary primary prevention in general practice		X			X

1. The EPA Cardio instrument, which is a set of indicators and related measures. The EPA Cardio instrument was based on a modified Delphi procedure to identify relevant indicators [14]. A total of 101 GPs from nine countries (80% of those invited) was involved in both rounds of this procedure. These countries were included again in this observational study, except for Spain which was added later. From an initial list of 650 indicators, 202 indicators were derived, from which 44 were rated valid (22%). Only indicators that scored high on necessity and feasibility in each of the country panels were included. These indicators covered lifestyle (8), clinical performance (27), and organisational aspects (9), and are incorporated in the following instruments: abstraction tool for a medical record audit in CHD patients; abstraction tool for a medical record audit in high risk patients; and an interview guide for an interview with the GP.

2. Health related lifestyles. In high risk and healthy patients, we will use questionnaires for specific aspects of lifestyle, including physical exercise (RAPA, 9 items) [15], diet (reduced REAP-S, 12 items) [16], and smoking (MID-SIZED Model, 8 items) [17].

3. Other measures on patients. In all patients (CHD, high risk and healthy), the questionnaires include items on demographic characteristics, healthcare use, chronic diseases, and patient experience with general practice (Europep new version, 23 items) [18]. In CHD and high risk patients, we added the EQ-5D (5 items + VAS scale) [19] and report on adherence to medication, if relevant (4 items) [20]. In CHD patients, we added the Patient Assessment of Chronic Illness Care (PACIC, 26 items) [21]

4. Practice engagement in quality improvement programs and education. The following items are measured in the interview with the GP or practice manager and in the practice questionnaire: practice engagement in cardiovascular quality improvement projects; practice engagement in public health projects concerning cardiovascular risk; practice engagement in other projects concerning cardiovascular risk management (structured lists of projects are used that are adapted to the national situation). A structured questionnaire for the GP/practice manager includes: questions on exposure to education and campaigns by nurses and GPs (five items); the average number of hours of continuing education on CVD and diabetes spent by health professionals in the practice in the previous two years (five items).

5. Other measures on practices. A written questionnaire is used to measure several practice characteristics: information process technology (EPA dimension, 11 items); organisation of chronic care and prevention (EPA dimension, 19 items); quality improvement (EPA dimension, eight items); practice staff tasks in cardiovascular care (five items for five types of staff); and practice size, in terms of listed patients and/or yearly attending patients. [22]

6. Innovative preventive services. A questionnaire has been drafted to explore the views of GPs and patients regarding what general practice could contribute to primary prevention of CVD. These questions are future-oriented, which implies that many doctors and patients are expected to have little or no experience with specific preventive activities. Also, the research evidence for effectiveness of the primary preventive services may be lacking or inconsistent.

All measures were translated systematically, using a forward and backward translation procedure and a testing phase with interviews. The final instruments were tested and adapted in a pilot project. In this project, the prototypes were tested in five countries in two practices each. This experience led to some minor adjustments in the audit forms and questionnaires. The measurements on primary prevention and the patient questionnaire for the 18 to 45 year age group was added after discussion to broaden the scope of the study: not just patients with established CVD (especially CHD) and patients at high risk, but also the generally healthy 18 to 45 year age group.

### Data-processing and data-analysis

In each of the participating countries, data will be entered into a database (Excel, SPSS, or other data management program). Some participants will make use of automated data-entry systems and other participants will use double entry of data to reduce errors. All frequency distributions will be checked for errors and the number of missing values will be noted. We will attempt to identify signs of responder fatigue, *e.g*., a series of questions that receive the same score. Also, we will examine the case mix of different practices in order to identify possible selection bias, caused by the sampling procedures.

Then, the findings will be described for each measure separately on a country-by-country basis. Appropriate summary measures will be used, such as mean and median values. The accuracy of the figures will be expressed in terms of 95% confidence intervals, taking into account that the data are nested at two levels: patients in practices, and practices in countries. A correction will be made for the nested data structure to avoid inappropriately inflated accuracy. We will examine qualitatively how the results of each country are related to the assessment of the quality indicators by the GP panel in that country.

Further statistical analysis of comparisons between subgroups in the project (defined by patients, practices, or countries) will take this nested data structure into account. Therefore, random coefficient regression models will be applied (linear or logistic, as appropriate). To reduce the possibility of chance capitalization, we will use p < 0.01 to indicate significance in explorative analyses and the conventional p < 0.05 in hypothesis-driven analyses.

### Time frame

The EPA Cardio project is planned from November 2005 to June 2009. The study described in this protocol is planned for March 2008 to April 2009.

## Competing interests

The authors declare that they have no competing interests.

## Authors' contributions

MW developed the overall outline of the EPA Cardio project and wrote the draft and final version of this study protocol. SL co-ordinated the development and pilot-testing of the measures. SC co-ordinated the international selection of performance indicators, which underly the EPA Cardio audit instrument. JvL contributed to the development and selection of measures. EV and RG are the project leaders of EPA Cardio. All authors critically assessed and approved this study protocol.

## Appendix 1 – Definitions

'Cardiovascular diseases' refer in this project to diseases due to atheroscleroris, such as angina pectoris, myocardial infarction, and stroke (CVA). Diabetes mellitus is also considered CVD by some experts, but this project does not focus on diabetes (except that it is recorded as co-morbidity).

'Cardiovascular risk factors' include age, gender, lifestyle factors (smoking, poor diet, overweight, physical inactivity, problematic use of alcohol), and clinical factors (diabetes, hypertension and serum cholesterol). The specific definitions of these factors, and the cut-off levels for high risk, vary across clinical guidelines. For instance, various blood pressure levels have been used to define hypertension. Increasingly, policy and practice focus on a patient's global risk rather than individual risk factors

'Cardiovascular risk management' is used broadly and includes the following target groups: the total population, particularly individuals with unhealthy life-styles; individuals who have high risk for CVD, which is defined in different ways and may be based on one or more risk factors; and patients who have established CVD.
